# Metabolic syndrome and high-sensitivity C-reactive protein co-modify the risk of coronary artery calcification

**DOI:** 10.3389/fcvm.2026.1759522

**Published:** 2026-03-30

**Authors:** Li-Yu Chen, Yen-Kung Chen, Vy-Khanh Nguyen, Chung-Kuan Wu

**Affiliations:** 1 Department of Family Medicine, Shin Kong Wu Ho-Su Memorial Hospital, Taipei, Taiwan; 2 Department of Nuclear Medicine and PET Center, Shin Kong Wu Ho-Su Memorial Hospital, Taipei, Taiwan; 3 School of Medicine, Fu Jen Catholic University, New Taipei, Taiwan; 4 College of Management, School of Health Care Administration, Taipei Medical University, Taipei, Taiwan; 5 College of Medical Science, School of Pharmacy, University of South Australia, Adelaide, SA, Australia; 6 Division of Nephrology, Department of Internal Medicine, Shin-Kong Wu Ho-Su Memorial Hospital, Taipei, Taiwan; 7 Division of Digital Informatics Management, Department of Digital Medicine, Shin Kong Wu Ho-Su Memorial Hospital, Taipei, Taiwan

**Keywords:** atherosclerosis, cardiovascular risk, coronary artery calcification, Hs-CRP, inflammation, metabolic syndrome

## Abstract

**Background:**

Coronary artery calcification (CAC) is a marker of subclinical atherosclerosis and is strongly associated with coronary artery disease (CAD). Metabolic syndrome (MetS) and high-sensitivity C-reactive protein (hs-CRP), an inflammatory marker, have each been linked to CAC, but their combined influence remains unclear.

**Methods:**

This cross-sectional study included 1948 adults undergoing health checkups and coronary calcium scoring via computed tomography. Participants were grouped by MetS status and hs-CRP levels (<0.3 vs. ≥0.3 mg/dL). Multivariate logistic regression analysis was used to evaluate associations between MetS, hs-CRP, and CAC, adjusting for age, sex, and clinical variables.

**Results:**

The cohort (mean age, 49 years; 78% male) was categorized into four groups: MetS(−)/low hs-CRP (74%), MetS(−)/high hs-CRP (10%), MetS(+)/low hs-CRP (12%), and MetS(+)/high hs-CRP (5%). The MetS(+)/high hs-CRP group had the highest Agatston score. CAC prevalence increased with the number of MetS components (from 44.5% to 100%). Among MetS components, high fasting glucose [adjusted odds ratio [aOR], 1.973; 95% confidence interval [CI], 1.526–2.551], hypertension (aOR, 1.674; 95% CI, 1.324–2.117), and high waist circumference (aOR, 1.492; 95% CI, 1.187–1.877) had the strongest associations with CAC. Elevated hs-CRP was independently associated with CAC (aOR, 1.631; 95% CI, 1.205–2.208), with a dose-response trend per 1 mg/dL increase (aOR, 1.360; 95% CI, 0.990–1.869). Compared to the MetS(−)/low hs-CRP group, the odds of CAC were highest in the MetS(+)/high hs-CRP group (aOR, 2.392; 95% CI, 1.470–3.891), followed by MetS(+)/low hs-CRP (aOR, 1.996; 95% CI, 1.426–2.795), and MetS(–)/high hs-CRP (aOR, 1.538; 95% CI, 1.067–2.218).

**Conclusions:**

MetS showed a stronger association with CAC than hs-CRP, while hs-CRP appeared to confer a modest incremental association.

## 1 Introduction

Coronary artery disease (CAD), the most common type of heart disease, poses a high risk for cardiovascular mortality. Risk factors for CAD included diabetes mellitus, hypertension, dyslipidemia, smoking, obesity, and family history of CAD. CAD results from arterial wall injury and inflammatory processes that lead to atherosclerosis and plaque calcification. Many studies have reported a strong correlation between the coronary artery calcium score (CACS) and CAD (1–3).

Metabolic syndrome (MetS), a cluster of cardiovascular risk factors, is increasingly prevalent. From 2011 to 2012 to 2017–2018, its prevalence rose from 37.6% to 41.8% (4). MetS was found to be associated with about a 1.5-fold increased risk of incident cardiovascular morbidity and mortality (5). A previous study showed that The risk of CACS increases with a greater number of MetS components and is further influenced by the specific combination of metabolic risk factors present (6). In addition, the prevalence of CACS increased with an increasing number of MetS components (7).

The most extensively investigated inflammatory biomarker of atherosclerotic cardiovascular disease is C-reactive protein (CRP), an acute-phase protein primarily synthesized by hepatocytes in response to cytokines such as interleukin-6 and tumor necrosis factor-alpha (8). High-sensitivity CRP (hs-CRP) is identified using a highly sensitive assay. The hs-CRP assay assesses cardiovascular risk by detecting low-grade inflammation more sensitively than traditional CRP assays. However, the association between hs-CRP and CACS in healthy individuals remains inconclusive (9, 10).

Patients with MetS tend to have higher hs-CRP levels (11, 12). While CACS is closely linked to MetS, the relationship between CAC and hs-CRP is under debate (13–16). Most previous investigations have examined MetS and hs-CRP as separate risk factors, leaving their combined impact insufficiently characterized. Therefore, this study aims to evaluate the joint effects of MetS and hs-CRP on CACS and to determine whether systemic inflammation confers additive risk beyond metabolic abnormalities alone.

## 2 Materials and methods

### 2.1 Study population

**Figure 1:**
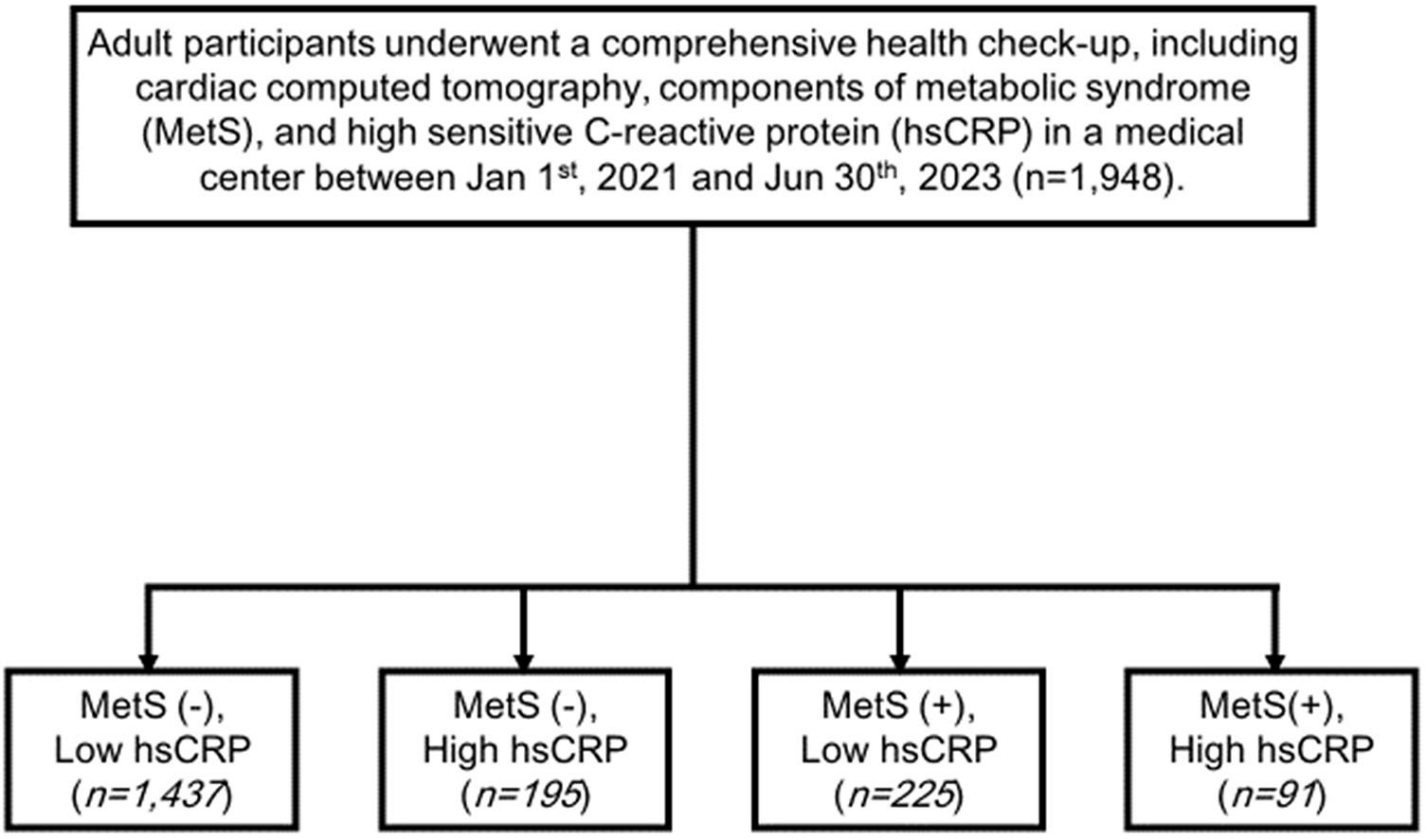
Flowchart of participant enrollment and classification based on metabolic syndrome (MetS) and hs-CRP levels.

A total of 1,948 adult participants who received health checkups at a medical center in northern Taiwan between January 1, 2021 and June 30, 2023 were enrolled in the study. The participants all had sufficient data available to diagnose MetS, and all underwent cardiac computed tomography to determine their Agatston score. The participants were divided into four groups according to their MetS status and hs-CRP level (cutoff: 0.3 mg/dL), as shown in Figure 1.

### 2.2 Collection of demographic and lab data and definition of metabolic syndrome (MetS)

Demographic data were obtained from the patients' medical records during entry to the study. The data included age and sex. Height, weight, waist circumference and resting blood pressure were measured. In addition, we collected laboratory data for the levels of hemoglobin, platelets, albumin, alanine transaminase, creatinine, sodium, potassium, calcium, low- and high-density lipoprotein-cholesterol, total cholesterol, triglycerides, fasting blood glucose, and hs-CRP after an 8-hour fast.

MetS was defined according to the National Cholesterol Education Program Adult Treatment Panel III criteria (17) as the presence of ≥3 of the following 5 criteria: (1) abdominal obesity based on waist circumference >80 cm for women and >90 cm for men; (2) HDL-cholesterol <40 mg/dL for men or <50 mg/dL for women; (3) fasting triglycerides ≥150 mg/dL; (4) systolic blood pressure (SBP) ≥130 mmHg or diastolic blood pressure (DBP) ≥85 mmHg or on treatment for hypertension; (5) fasting blood glucose >100 mg/dL.

### 2.3 Measurement

#### 2.3.1 High-sensitivity C-reactive protein (hs-CRP)

The measurement method of hs-CRP is based on immunoturbidimetry, and the instrument used for this purpose is the LABOSPECT 008AS automatic biochemical analyzer (Hitachi, Tokyo, Japan). The cutoff value for hs-CRP in this study was 0.3, regardless of diabetic mellitus. Previous studies in both diabetic and non-diabetic populations support the use of 0.3 mg/dL as a cardiovascular risk cutoff (18–21).

#### 2.3.2 Calcium score for coronary arteries

Coronary calcium scoring was performed using a Revolution CT scanner (GE Healthcare, Chicago, IL, USA). The Agatston score, calculated using a semi-automated method, multiplies the area of calcified lesions by a weighted density score based on peak attenuation. A radiologist blinded to patient data verified all results. The Agatston score corresponds to the degree of calcification severity. The presence of CAC was defined as Agatston score ≥1.

### 2.4 Statistical analysis

Continuous variables are presented as the mean with standard deviation. Categorical variables were presented as counts with percentages. The normality of continuous variables was assessed using the Kolmogorov–Smirnov test. Categorical variables in four groups were compared using the chi-square test, and continuous variables in four groups were compared using one-way ANOVA or the Kruskal–Wallis test, depending on whether the data distribution was normal. Binary logistic regression analysis was conducted for each component of MetS and hs-CRP to assess the crude odds ratio (cOR) for CAC. In addition, logistic regression analysis was used to determine the cOR for CAC between the four groups. Models were adjusted for age, sex, and clinical variables including hemoglobin, platelets, albumin, alanine transaminase (ALT), and creatinine levels. A two-tailed *p* < 0.05 was considered statistically significant.

## 3 Results

### 3.1 Comparison of baseline clinical and lab data among four groups according to the presence of MetS and level of hs-CRP

Of the 1,948 adult participants (mean age, 49 years), 1,513 (78%) were men and 435 (22%) were women. The proportion of participants in the cohort with MetS was 19% among men and 9% among women. For data analysis, the cohort was divided into 4 groups as follows: without MetS with low hs-CRP (*n* = 1,437, 74%); without MetS with high hs-CRP, (*n* = 195, 10%); with MetS with low hs-CRP, (*n* = 225, 12%); and with MetS with high hs-CRP, (*n* = 91, 5%).

Compared to the other groups, participants with MetS and high hs-CRP had significantly higher waist circumference; levels of fasting blood glucose, hemoglobin, platelets, alanine transaminase, and creatinine; and Agatston score but the lowest HDL level. Participants with MetS and low hs-CRP were significantly older and had the highest blood pressure and the highest levels of serum triglycerides and serum albumin. The group of participants without MetS and low hs-CRP had the highest proportion of females. No significant differences between the four groups were observed with respect to sodium, potassium, calcium, total cholesterol, and LDL levels (Table 1).

**Table 1:** Clinical and laboratory characteristics according to metabolic syndrome status and hs-CRP levels.

Variables	MetS (-), Low hsCRP	MetS (-), High hsCRP	MetS (+), Low hsCRP	MetS(+), High hsCRP	*P*
	(*n* = 1,437)	(*n* = 195)	(*n* = 225)	(*n* = 91)	
Sex, female (%)	353 (24.6)	47 (24.1)	22 (9.8)	13 (14.3)	<0.001^†^
Age (years)	48.75 ± 9.46	48.13 ± 10.55	51.36 ± 9.33	48.55 ± 8.42	0.001^§^
MetS Components					
Waist circumference (cm)	81.58 ± 8.76	85.85 ± 10.24	92.61 ± 7.36	96.29 ± 9.92	<0.001^§^
SBP (mmHg)	118.29 ± 15.79	121.00 ± 16.83	133.66 ± 17.28	130.48 ± 18.19	<0.001^§^
DBP (mmHg)	75.79 ± 9.11	77.79 ± 10.51	84.83 ± 10.09	84.59 ± 12.64	<0.001^§^
Fasting blood glucose (mg/dL)	92.76 ± 14.05	96.57 ± 22.08	106.28 ± 21.85	114.00 ± 36.56	<0.001^§^
Triglyceride (mg/dL)	104.85 ± 57.00	114.33 ± 59.85	216.48 ± 136.05	210.81 ± 141.70	<0.001^§^
HDL (mg/dL)	58.22 ± 14.76	53.56 ± 12.51	42.75 ± 8.98	41.20 ± 9.65	<0.001^§^
Lab data					
Hb (g/dL)	14.60 ± 1.47	14.71 ± 1.54	15.22 ± 1.30	15.35 ± 1.49	<0.001^§^
Platelets (10^3^/µL)	255.82 ± 57.92	275.30 ± 68.31	263.02 ± 59.89	276.00 ± 59.39	<0.001^§^
Albumin (g/dL)	4.53 ± 0.22	4.48 ± 0.25	4.55 ± 0.23	4.50 ± 0.24	0.006^#^
GPT/ALT (U/L)	27.40 ± 24.05	31.48 ± 25.58	39.48 ± 27.50	41.00 ± 22.08	<0.001^§^
Creatinine (mg/dL)	0.87 ± 0.26	0.91 ± 0.48	0.90 ± 0.17	0.92 ± 0.23	0.009^§^
Na (mmol/L)	142.32 ± 2.30	141.87 ± 2.54	141.85 ± 2.41	141.94 ± 2.29	0.190^§^
K (mmol/L)	4.06 ± 0.35	4.03 ± 0.32	4.04 ± 0.39	3.96 ± 0.39	0.299^§^
Ca (mg/dL)	9.09 ± 0.42	9.09 ± 0.46	9.17 ± 0.44	9.09 ± 0.44	0.451^§^
Cholesterol (mg/dL)	202.14 ± 37.04	202.09 ± 40.64	201.80 ± 38.57	203.74 ± 43.68	0.943^§^
LDL (mg/dL)	127.25 ± 33.03	129.37 ± 37.48	126.63 ± 32.92	129.71 ± 40.12	0.994^§^
hsCRP (mg/dL)	0.08 ± 0.07	0.77 ± 0.75	0.12 ± 0.07	0.63 ± 0.47	<0.001^§^
Agatston score	74.49 ± 367.22	82.00 ± 264.96	152.86 ± 387.11	185.10 ± 725.17	<0.001^§^

Data are expressed as *n* (%) for categorical variables and mean ± standard deviation for continuous variables. BMI, body mass index; SBP, systolic blood pressure; DBP, diastolic blood pressure; HDL, high-density lipoproteins; LDL, low-density lipoproteins; GPT/ALT, alanine transaminase.

^#^ One-way analysis of variance (ANOVA)

^§^ Kruskal–Wallis test

^†^ Chi-square test.

### 3.2 Association of MetS components and hs-CRP with coronary artery calcification

**Figure 2:**
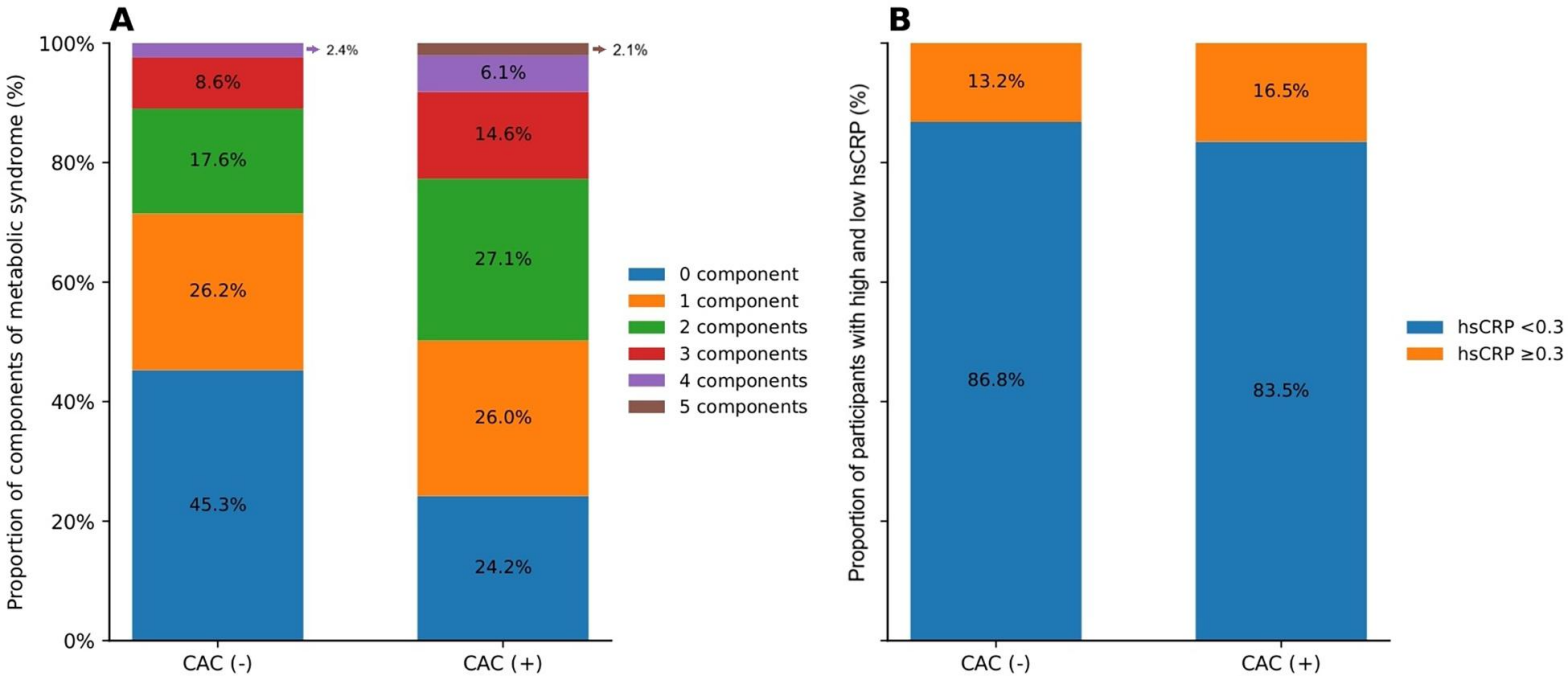
**(A)** Distribution of MetS components among participants with and without CAC. The bar graph illustrates the proportion of participants with 0–5 metabolic syndrome (MetS) components, stratified by the presence of coronary artery calcification (CAS). **(B)** Distribution of hsCRP levels among participants with and without CAC. The bar graph presents the proportion of participants with high-sensitivity C-reactive protein (hsCRP) levels <0.3 mg/dL and ≥0.3 mg/dL, stratified by the presence of coronary artery calcification (CAC). Among those without CAC (*n* = 1,076), 934 (86.8%) had hsCRP <0.3 mg/dL and 142 (13.2%) had hsCRP ≥0.3 mg/dL. In contrast, among participants with CAC (*n* = 872), 728 (83.5%) had hsCRP <0.3 mg/dL and 144 (16.5%) had hsCRP ≥0.3 mg/dL.

When grouped according to the number of MetS criteria met, the percentage of patients with a positive Agatston score increased with the number of MetS criteria components (Figure 2A). Among participants with 1, 2, 3, 4, and 5 MetS components, the prevalence of CAC was 44.5%, 55.5%, 57.9%, 67.0%, and 100%, respectively.

The risk of CAC for each MetS criterion is shown in Table 2. For participants meeting the waist circumference criterion, the crude odds ratio (cOR) for CAC was 1.479 (95% CI, 1.221–1.791) compared to those who did not. After adjusting for age, sex or age, sex, and hemoglobin, platelets, albumin, ALT, and creatinine, participants meeting the waist circumference criterion still had a higher adjusted odds ratio (aOR) for CAC (1.516, 1.492; 95% CI, 1.213–1.896; 1.187–1.877, respectively). Compared to participants with normal blood pressure, participants with hypertension had cOR and aOR for CAC in model 1 and 2 of 2.379, 1.711, and 1.674 (95% CI, 1.948–2.905; 1.359–2.154; 1.324–2.117), respectively. For participants meeting the criteria of high fasting blood glucose, the cOR and aOR in models 1 and 2 for CAC were 3.713, 2.033, and 1.973 (95% CI, 2.966–4.649; 1.578–2.619; 1.526–2.551) compared to those without. For participants meeting the serum triglyceride criterion, the cOR and aOR in for CAC for models 1 and 2 were 1.641, 1.477, and 1.417 (95% CI, 1.337–2.014; 1.167–1.870; 1.114–1.803). For participants with low HDL, the cOR and aOR in for CAC in models 1and 2 were 1.301, 1.409, and 1.368 (95% CI, 1.010–1.674; 1.056–1.881; 1.023–1.829) respectively, compared to those with high HDL.

**Table 2:** Logistic regression analysis of metabolic syndrome components against coronary arterial calcification.

MS components	Crude			Model 1			Model 2		
	cOR	95% CI	*p*	aOR	95% CI	*p*	aOR	95% CI	*p*
High waist circumference	1.479	1.221–1.791	<0.001	1.516	1.213–1.896	<0.001	1.492	1.187–1.877	<0.001
Hypertension	2.379	1.948–2.905	<0.001	1.711	1.359–2.154	<0.001	1.674	1.324–2.117	<0.001
High fasting blood glucose	3.713	2.966–4.649	<0.001	2.033	1.578–2.619	<0.001	1.973	1.526–2.551	<0.001
High triglycerides	1.641	1.337–2.014	<0.001	1.477	1.167–1.870	0.001	1.417	1.114–1.803	0.005
Low HDL	1.301	1.010–1.674	0.042	1.409	1.056–1.881	0.020	1.368	1.023–1.829	0.035
High hsCRP	1.301	1.012–1.673	0.040	1.644	1.223–2.212	0.001	1.631	1.205–2.208	0.002
hsCRP per 1 mg/dL increase	1.243	0.957–1.616	0.103	1.356	0.987–1.862	0.060	1.360	0.990–1.869	0.058

Each component was categorized using cut-off points, as follows: high waist circumference, ≥90 cm for males, ≥80 cm for females; presence of hypertension, SBP ≥130 mm Hg and DBP ≥85 mmHg; high fasting glucose, ≥100 mg/dL; high triglycerides ≥150 mg/dL; low HDL, <40 mg/dL for males, <50 mg/dL for females; high hsCRP, ≥0.3 mg/dL; hsCRP per 1 mg/dL increase: dose-response analysis of hsCRP level with CAC risk.

**Crude:** Univariate model.

**Model 1:** Multivariate model adjusted by age and sex.

**Model 2:** Multivariate model adjusted by age, sex, Hb, platelets, Albumin, GLP/ALT and Creatinine in **Crude model**.

A higher proportion of participants with high hs-CRP was observed among those with a positive Agatston score compared to those without (Figure 2B). For participants with high hs-CRP, the cOR and aORs for CAC in models 1and 2 were 1.301, 1.644, and 1.631 (95% CI, 1.012–1.673; 1.223–2.212; 1.205–2.208), respectively, compared to those with low hs-CRP. In addition, each 1 mg/dL increase in hs-CRP increased the risk for CAC by 1.243, 1.356, and 1.360, respectively, in crude and adjusted models 1 and 2 (95% CI, 0.957–1.616; 0.987–1.862; 0.990–1.869) (Table 2).

### 3.3 CAC risk in healthy participants according to the presence of MetS and level of hs-CRP

Binary logistic regression analysis showed that the cOR for CAC was 1.248, 2.692, 2.126 (95% CI, 0.924–1.686; 2.007–3.609; 1.381–3.272) for participants without MetS and high CRP, with MetS and low CRP, and with MetS and high CRP, respectively, compared to those without MetS and low hs-CRP (Table 3). After adjustment for age and sex (model 1) or age, sex and significant variables in Table 1 (model 2), the aORs for CAC were 1.533, 1.538 (95% CI: 1.068–2.200, 1.067–2.218) for those without MetS and high CRP; 2.073, 1.996 (95% CI: 1.487–2.889, 1.426–2.795) for those with MetS and low CRP; and 2.411, 2.392 (95% CI: 1.496–3.885, 1.470–3.891) for those with MetS and high CRP.

**Table 3:** Binary logistic regression analysis of CAC risk.

Outcomes	Crude			Model 1			Model 2		
	cOR	95% CI	*p*	aOR	95% CI	*p*	aOR	95% CI	*p*
MetS(−), low hsCRP	1			1			1		
MetS(−), high hsCRP	1.248	0.924–1.686	0.148	1.533	1.068–2.200	0.020	1.538	1.067–2.218	0.021
MetS(+), low hsCRP	2.692	2.007–3.609	<0.001	2.073	1.487–2.889	<0.001	1.996	1.426–2.795	<0.001
MetS(+), high hsCRP	2.126	1.381–3.272	0.001	2.411	1.496–3.885	<0.001	2.392	1.470–3.891	<0.001

**Model 2:** Adjusted for age and sex (aOR, adjusted odd ratio).

**Model 3:** Adjusted for age, sex, Hb, platelets, albumin, GLP/ALT, and creatinine in Table 1.

## 4 Discussion

We observed that participants with MetS and high hs-CRP had the largest waist circumference and highest levels of fasting blood glucose, hemoglobin, platelets, ALT, and creatinine and lowest HDL levels. The fraction of participants with MetS was higher among those with CAC than in those without, and each component of MetS was independently associated with a risk of CAC. Of the MetS components, high fasting blood glucose posed the highest risk for CAC, followed by high blood pressure, high waist circumference, high triglycerides, and low HDL. In addition, a higher proportion of participants with elevated hs-CRP was observed among those with CAC, and hs-CRP remained an independent risk factor for CAC after adjustment for age, sex, BMI, blood pressure, fasting glucose, lipid levels, creatinine, ALT, hemoglobin, and platelets in our study population. MetS and high hs-CRP co-modified the risk of CAC in the study cohort.

The highest waist circumference, fasting blood glucose level, and platelet count and lowest HDL level were observed among adults with MetS and high hs-CRP, likely reflecting the interaction between metabolic dysregulation and inflammation. This environment promotes obesity, insulin resistance, endothelial dysfunction, and organ impairment. Obesity and endothelial dysfunction may increase platelet activation through cytokines and growth factors (22). In addition, MetS commonly leads to non-alcoholic fatty liver disease, which elevates liver enzyme levels (23). In turn, elevated liver enzyme levels contribute to the development and progression of MetS through inflammation and oxidative stress (24, 25). Similarly, an association between MetS and CKD is biologically plausible, as visceral obesity is strongly linked to insulin resistance, and markers of visceral obesity may serve as sensitive predictors of kidney disease (26). Furthermore, the environment of excess adipose tissue provides sources of inflammatory and immunomodulatory factors that also exacerbate MetS (27–29). This pro-inflammatory environment further accelerates renal dysfunction, leading to elevated creatinine levels. We also observed the highest level of hemoglobin among adults with MetS and high hs-CRP. These findings were also observed in several studies (30–35); however, the ways in which these three parameters interact remains unknown. A plausible explanation is that the hemoglobin concentration correlates with some components of MetS (36).

Our results show a significant and positive correlation between the number of MetS components and the prevalence of CAC. We also observed that every component of MetS posed a significant risk for CAC, a result contradicted by some studies. In our study, a high fasting blood glucose level posed the highest risk for CAC of all MetS components. A Korean study showed that impaired fasting blood glucose (especially ≥110 mg/dL) is an independent risk factor for CAC (37). A cohort study noted that young adults with elevated insulin resistance were at a greater risk of developing CAC in middle age (38). A trend of higher CAC was noted among individuals with diabetes as compared to those without (39). However, another study using a community-based cohort without diabetes found that CACS was not associated with impaired fasting glucose (100–125 mg/dL), and hypertension was the second-highest risk factor for CAC among the MetS components (40). A Korean study showed that blood pressure correlated positively with the presence of CAC, even in those with pre-hypertension. This trend was similar even among young and low-risk individuals (41). Hypertension is strongly linked to increased CAC levels (42). However, a Korean study reports that CAC was not significantly associated with systolic or diastolic blood pressure or waist circumference (43).

Our study found that high waist circumference was the third highest risk factor for CAC among MetS components. A multi-ethnic study reports that waist circumference was associated with CAC as determined using the spatially-weighted calcium score (44). Of the remaining MetS components, triglycerides ranked fourth for CAC risk. A prospective cohort study showed that hypertriglyceridemia was associated with subclinical atherosclerosis and vascular inflammation (45). A study of pooled data obtained from 6,544 subjects showed that the serum triglyceride level was associated with CAC progression (46). Low HDL was a risk factor for CAC in this study, although the odds ratio for CAC was the lowest of all the MetS components. A comparative study demonstrated that higher HDL levels were associated with reduced coronary calcification and a lower likelihood of having any calcified disease (47). However, this result was considered controversial in a systematic review (48).

Our study provides evidence supporting an auxiliary role for hs-CRP in coronary calcification. An analysis of 321 individuals without previous cardiovascular disease from the Framingham Heart Study identified a correlation between elevated C- CRP levels and higher Agatston scores (15). However, the risk of hs-CRP for CAC remains unknown. A systematic review and meta-analysis concluded that an elevated CRP level did not serve as a reliable prognostic marker for the incidence or progression of CAC (49).

Recently, the concept of cardiovascular–kidney–metabolic (CKM) syndrome has been proposed to describe a pathophysiological continuum linking metabolic disorders, systemic inflammation, and the development of cardiovascular and renal diseases (50, 51). Within this framework, obesity and adipose tissue dysfunction contribute to metabolic abnormalities and promote chronic low-grade inflammation, oxidative stress, and vascular dysfunction, which together accelerate atherosclerotic processes. While the CKM framework emphasizes the interconnected roles of metabolic dysregulation and inflammation in the development of cardiovascular disease, it does not delineate the relative contribution of these pathways to atherosclerotic burden. Our findings are consistent with this integrated model. In the present study, both metabolic syndrome and elevated hs-CRP were independently associated with the presence of coronary artery calcification, a well-established marker of subclinical atherosclerosis. Moreover, individuals with concomitant metabolic syndrome and elevated hs-CRP exhibited the highest likelihood of CAC, suggesting that metabolic dysregulation and systemic inflammation may exert additive effects on vascular calcification. Importantly, our results further suggest that metabolic syndrome represents the primary determinant of CAC, whereas systemic inflammation, reflected by elevated hs-CRP, confers an additional effect that amplifies this risk. These observations support the CKM syndrome framework by providing population-based evidence that the coexistence of metabolic abnormalities and inflammatory activation is associated with a greater burden of subclinical coronary atherosclerosis. Taken together, our results highlight the importance of considering metabolic and inflammatory pathways simultaneously when evaluating early cardiovascular risk.

### 4.1 Clinical implications

The interaction between MetS and the inflammatory biomarker hs-CRP in CAC risk carries important clinical implications. First, the presence of MetS should be a central focus in CAC risk assessment, particularly in individuals with multiple MetS components. Second, elevated hs-CRP may serve as an additional risk stratification tool, especially in the context of MetS. These findings suggest that incorporating a detailed analysis of MetS components and analysis of inflammatory markers such as hs-CRP could improve cardiovascular risk prediction.

### 4.2 Study limitations and future directions

Despite the important insights provided by this study, several limitations should be acknowledged. First, the cross-sectional design prevents us from establishing causality. Second, the participants were all individuals undergoing health checkups at a medical center in northern Taiwan, potentially introducing selection bias and limiting the generalizability of the findings to other populations. Third, we used only one type of MetS criteria. However, a past study comparing the three types of MetS criteria (International Diabetes Federation, National Cholesterol Education Program Adult Treatment Panel III, and European Group for the Study of Insulin Resistance) reported no significant difference in the ability of the three MetS criteria sets to predict CAC (52).

In addition, lifestyle-related factors such as smoking status, physical activity, dietary habits, and medication use were not comprehensively evaluated in this study, which may have resulted in residual confounding. Additionally, hs-CRP measurements were based on a single assessment, which may not capture its variability over time. Future research should consider a longitudinal design to explore the causal relationships between MetS, hs-CRP, and CAC and to extend investigations to multicenter cohorts for broader applicability.

## 5 Conclusion

This study demonstrates that MetS is the primary determinant of CAC, with elevated hs-CRP imposing an additive effect that further amplifies this risk. While MetS components such as abdominal obesity, elevated blood pressure, elevated fasting glucose, and dyslipidemia directly contribute to the pathophysiology of calcification, hs-CRP appears to increase the inflammatory milieu, thereby augmenting the calcification process. These findings highlight the dominant role of MetS in promoting CAC, with hs-CRP serving as a complementary factor rather than a primary driver. A comprehensive evaluation strategy that integrates metabolic and inflammatory markers may increase the precision of CAC risk prediction and help mitigate the cardiovascular risks associated with calcification.

## Data Availability

The raw data supporting the conclusions of this article will be made available by the authors, without undue reservation.
