# Supplementary material for: Metabolic syndrome and high-sensitivity C-reactive protein co-modify the risk of coronary artery calcification
Source: Front Cardiovasc Med. 2026 Mar 30;13:1759522. doi: 10.3389/fcvm.2026.1759522 (PMC13070749; doi:10.3389/fcvm.2026.1759522)
Supplement: Supplementary file 1 [file Table1.docx]

**Supplementary Table 1.** Baseline characteristics of participants on examination presence/absence of CAC (N = 1948), calcium score grade (N = 872)

|  | **CAC (-)** | **CAC (+)** | *p* | **Minimal** | **Mild** | **Mod** | **Severe** | *p* |
| --- | --- | --- | --- | --- | --- | --- | --- | --- |
| Sample size | 1076 | 872 |  | 215 | 368 | 173 | 116 |  |
| Sex, female (%) | 341 (31.7) | 94 (10.8) | <0.001^†^ | 30 (14.0) | 37 (10.1) | 22 (12.7) | 5 (4.3) | 0.043^†^ |
| Age | 45.2 ± 8.3 | 53.6 ± 8.9 | <0.001^#^ | 49.8 ± 7.7 | 53.0 ± 8.2 | 55.4 ± 8.9 | 59.7 ± 9.3 | <0.001^§^ |
| **Components of MetS** |  | | | | | |  |  |
| Waist circumference | 82.1 ± 10.2 | 86.2 ± 8.9 | <0.001^#^ | 86.3 ± 9.4 | 85.8 ± 8.9 | 85.9 ± 8.7 | 87.9 ± 8.3 | 0.119^§^ |
| SBP (mmHg) | 117.1 ± 15.8 | 125.6 ± 17.3 | <0.001^#^ | 123.6 ± 16.2 | 124.9 ± 16.8 | 126.2 ± 17.7 | 130.9 ± 19.1 | 0.008^§^ |
| DBP (mmHg) | 75.7 ± 10.0 | 79.5 ± 9.9 | <0.001^#^ | 79.5 ± 10.2 | 79.6 ± 9.7 | 79.6 ± 10.4 | 79.4 ± 9.4 | 0.895^§^ |
| Fasting blood glucose | 92.1 ± 14.2 | 100.1 ± 22.2 | <0.001^#^ | 96.1 ± 15.6 | 99.4 ± 19.9 | 101.8 ± 22.6 | 107.4 ± 33.7 | 0.002^§^ |
| Triglycerides | 113.5 ± 75.6 | 136.1 ± 96.4 | <0.001^#^ | 136.4 ± 84.9 | 130.2 ± 69.1 | 159.1 ± 155.4 | 120.4 ± 67.2 | 0.153^§^ |
| HDL | 57.6 ± 15.9 | 52.2 ± 13.2 | <0.001^#^ | 51.6 ± 13.3 | 53.2 ± 12.9 | 51.5 ± 13.4 | 51.5 ± 13.2 | 0.266^§^ |
| **Lab data** |  | | | | | |  |  |
| Hb | 14.5 ± 1.5 | 14.9 ± 1.4 | <0.001^#^ | 15.0 ± 1.4 | 14.9 ± 1.3 | 14.8 ± 1.52 | 14.9 ± 1.6 | 0.557^§^ |
| Platelet | 263.1 ± 58.7 | 255.1 ± 60.6 | 0.004^#^ | 261.3 ± 61.9 | 255.8 ± 56.6 | 260.1 ± 63.3 | 234.2 ± 62.9 | 0.001^§^ |
| Albumin | 4.5 ± 0.2 | 4.5 ± 0.2 | 0.898* | 4.5 ± 0.2 | 4.5 ± 0.2 | 4.5 ± 0.2 | 4.4 ± 0.3 | 0.038^¶^ |
| GPT/ALT | 28.4 ± 26.2 | 31.6 ± 23.2 | <0.001^#^ | 32.4 ± 20.5 | 32.3 ± 26.6 | 29.6 ± 18.7 | 31.1 ± 22.3 | 0.696^§^ |
| BUN | 13.4 ± 3.6 | 14.7 ± 5.7 | <0.001^#^ | 13.9 ± 3.4 | 14.0 ± 3.6 | 15.2 ± 5.6 | 17.2 ± 11.2 | 0.002^§^ |
| Creatinine | 0.8 ± 0.2 | 0.9 ± 0.7 | <0.001^#^ | 0.9 ± 0.2 | 0.9 ± 0.2 | 0.9 ± 0.5 | 1.1 ± 1.7 | 0.105^§^ |
| Na | 142.1 ± 2.1 | 142.3 ± 2.5 | <0.001^#^ | 142.6 ± 2.3 | 142.5 ± 2.4 | 141.6 ± 2.4 | 141.9 ± 2.9 | 0.088^§^ |
| K | 4.0 ± 0.3 | 4.1 ± 0.4 | 0.170^#^ | 4.0 ± 0.4 | 4.0 ± 0.4 | 4.1 ± 0.4 | 4.1 ± 0.4 | 0.129^§^ |
| Ca | 9.0 ± 0.4 | 9.1 ± 0.4 | <0.001^#^ | 9.1 ± 0.5 | 9.1 ± 0.4 | 9.2 ± 0.4 | 9.2 ± 0.5 | 0.234^§^ |
| Cholesterol | 201.6 ± 34.7 | 202.8 ± 41.5 | 0.904^#^ | 208.3 ± 41.8 | 206.6 ± 38.4 | 200.9 ± 46.9 | 183.5 ± 36.1 | <0.001^§^ |
| LDL | 126.3 ± 30.8 | 128.9 ± 37.1 | 0.211^#^ | 134.9 ± 39.6 | 132.6 ± 34.5 | 124.8 ± 39.9 | 112.6 ± 30.9 | <0.001^§^ |
| Agatston score | 0 ± 0 | 202.9 ± 561.5 | <0.001^#^ | 3.9 ± 2.8 | 38.9 ± 24.8 | 208.44 ± 85.1 | 1083.3 ± 1200.5 | <0.001^§^ |

Data are expressed as n (%) for categorical variables and mean ± standard deviation for continuous variables. CAS, coronary artery calcification score, BMI, body mass index; SBP, systolic blood pressure; DBP, diastolic blood pressure; HDL, high-density lipoproteins; LDL, low-density lipoproteins; GPT/ALT, alanine transaminase

*One-sample t-test #Mann-Whitney U test ^†^Chi-square test ^¶^ANOVA ^§^Kruskal–Wallis test

**Calcification scoring: 0, none; 1–10, minimal; 11–100, mild; 101–400, moderate; > 400, severe**
