# Supplementary material for: Metabolic syndrome and high-sensitivity C-reactive protein co-modify the risk of coronary artery calcification
Source: Front Cardiovasc Med. 2026 Mar 30;13:1759522. doi: 10.3389/fcvm.2026.1759522 (PMC13070749; doi:10.3389/fcvm.2026.1759522)
Supplement: Supplementary file 2 [file Presentation1.pptx]

## Slide 1
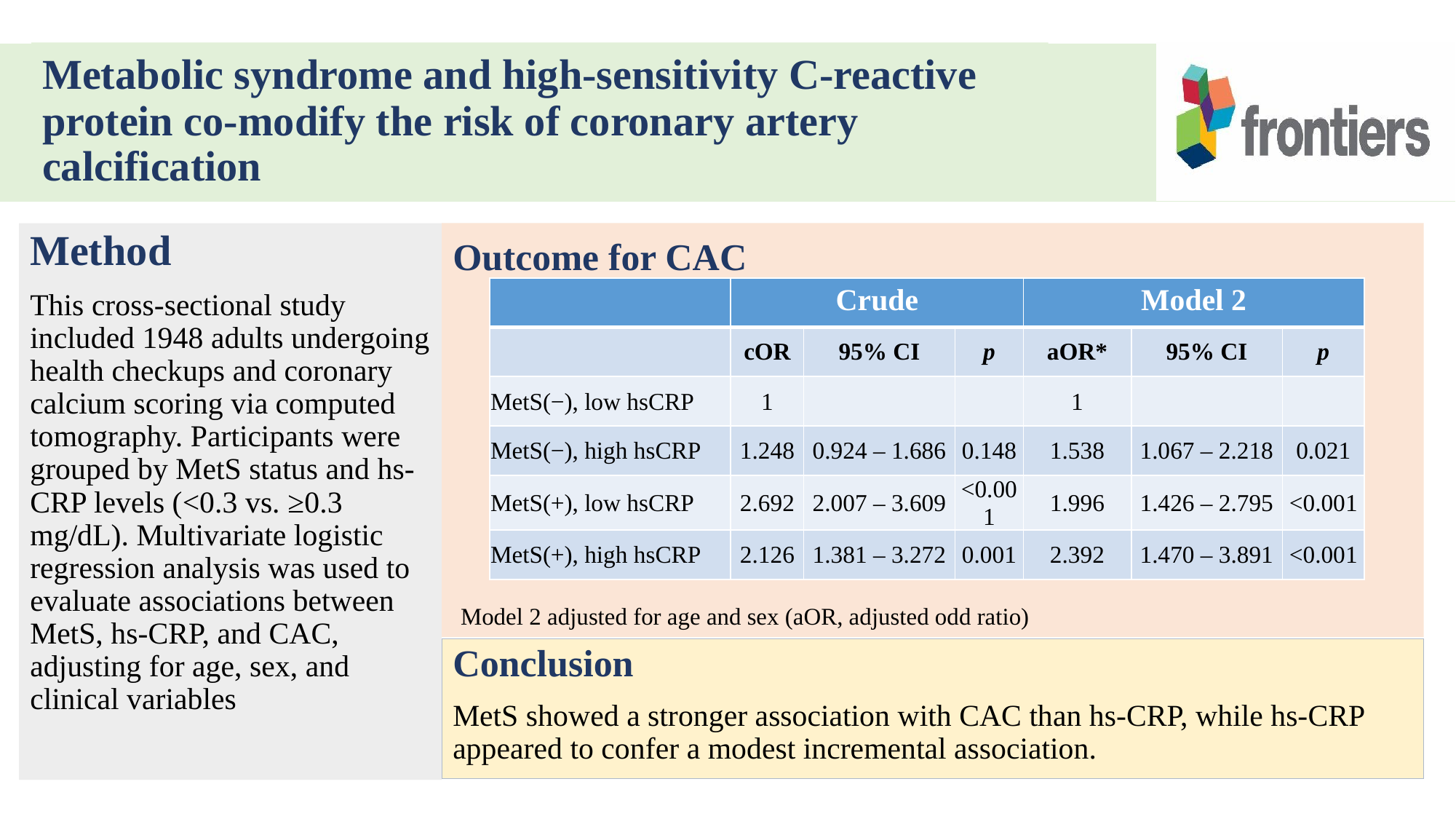

# Metabolic syndrome and high-sensitivity C-reactive protein co-modify the risk of coronary artery calcification
Method
This cross-sectional study included 1948 adults undergoing health checkups and coronary calcium scoring via computed tomography. Participants were grouped by MetS status and hs-CRP levels (<0.3 vs. ≥0.3 mg/dL). Multivariate logistic regression analysis was used to evaluate associations between MetS, hs-CRP, and CAC, adjusting for age, sex, and clinical variables
Outcome for CAC
 Model 2 adjusted for age and sex (aOR, adjusted odd ratio)
| | Crude | | | Model 2 | | |
| --- | --- | --- | --- | --- | --- | --- |
| | cOR | 95% CI | p | aOR\* | 95% CI | p |
| MetS(−), low hsCRP | 1 | | | 1 | | |
| MetS(−), high hsCRP | 1.248 | 0.924 – 1.686 | 0.148 | 1.538 | 1.067 – 2.218 | 0.021 |
| MetS(+), low hsCRP | 2.692 | 2.007 – 3.609 | <0.001 | 1.996 | 1.426 – 2.795 | <0.001 |
| MetS(+), high hsCRP | 2.126 | 1.381 – 3.272 | 0.001 | 2.392 | 1.470 – 3.891 | <0.001 |
Conclusion
MetS showed a stronger association with CAC than hs-CRP, while hs-CRP appeared to confer a modest incremental association.
